# Editorial: Reviews in microbiome in health & disease

**DOI:** 10.3389/fcimb.2024.1423386

**Published:** 2024-05-27

**Authors:** Suhana Chattopadhyay, Leena Malayil

**Affiliations:** Department of Global, Environmental, and Occupational Health, School of Public Health, University of Maryland, College Park, MD, United States

**Keywords:** gut microbiome, diseases, homeostasis, dysbiosis, reviews

The gut microbiome, a complex and diverse community inhabiting the gastrointestinal tract, is vital for maintaining health and impacting susceptibility to numerous diseases. Its interactions with the host are diverse, from aiding digestion and regulating immunity to influencing metabolism and protecting against pathogens. These interactions are pivotal in the development of chronic diseases and autoimmune conditions. Firstly, the gut microbiome plays a significant role in digesting complex carbohydrates and fibers, producing short-chain fatty acids crucial for gut health. Therefore, any disruption to the normal gut microflora could contribute to the development of obesity and metabolic disorders (Liu et al., 2021). Secondly, the gut microbiome produces neurotransmitters and neuroactive compounds like serotonin and gamma-aminobutyric acid, which can impact the mood, behavior, and cognitive function of the host. Imbalances in the gut microbiome have been associated with mental health disorders like anxiety and depression (Xiong et al., 2023). Thirdly, the gut microbiome acts as a protective barrier against harmful pathogens by competing for resources and producing antimicrobial compounds. Dysbiosis, an imbalance in the gut microbiota, has been linked to various chronic diseases including inflammatory bowel diseases, type 2 diabetes, cardiovascular diseases, and autoimmune conditions (Tsai et al., 2021). Fourthly, evidence suggests that the gut microbiome plays a key role in the development of allergies and autoimmune diseases (Xu et al., 2019). Early exposure to diverse microbes is believed to contribute to proper immune system training, thereby reducing the risk of hypersensitivity reactions and autoimmune responses.

Overall, the gut microbiome is a dynamic and essential component of human health. Its role extends beyond digestion to influence immune function, metabolism, mental health, and protection against diseases. Understanding the intricate interactions between the host and its microbiome opens avenues for therapeutic interventions and preventive strategies, highlighting the significance of maintaining a balanced and diverse gut microbial community for overall well-being. This Research Topic emphasizes several reviews that underscore the critical role of the gut microbiome in maintaining a delicate balance between health and disease.

While it is known that dysbiosis in the gut microbiome is considered a key factor in the host’s health and disease development through the microbiota-gut-brain (MGB) axis and the gut-lung axis, less is known about its impact extending to various conditions, including autism spectrum disorder (ASD) and attention-deficit/hyperactivity disorder (ADHD), or cystic fibrosis. One such review discusses how scientific evidence suggests that imbalances in the gut microbiome contribute to these disorders, highlighting the importance of the MGB axis in the treatment of ADHD and ASD (Kwak et al.). This review outlines the role of gut microbial colonization in early life and its connection to neurodevelopmental disorders like ASD/ADHD pathogenesis, emphasizing a promising therapeutic approach using psychobiotics and fecal microbial transplantation (FMT). Another review summarizes the impacts of the gut-lung axis on cystic fibrosis pathophysiology, highlighting the impact of cystic fibrosis transmembrane conductance regulator (CFTR) modulators in pulmonary and digestive microbiomes (Lussac-Sorton et al.). Albeit somewhat inconsistent, the review summarizes studies suggesting CFTR modulators that promote increased bacterial diversity with a reduction in conventional cystic fibrosis pathogens in the respiratory system and an increase in anti-inflammatory bacteria in the gut. This article showcases the use of CFTR modulators in the management of cystic fibrosis specifically for younger patients.

Furthermore, two other reviews highlight the importance of understanding the relationship between intestinal microbiome and surgical procedures. The first review by Ma et al. provides an overview of the pivotal role that early-life intestinal microflora provides in determining human health including the effects of multiple influencers like delivery mode, gestational age, and feeding method. The review highlights the importance of the maternal-infant symbiotic relationship in shaping an infant’s gut microbiota, along with raising concerns about cesarean section’s impact on the neonatal gut microbiome, emphasizing the need for long-term follow-up studies. Additionally, the review also explores natural and artificial reconstruction of intestinal flora in infants, intending to prevent and/or treat neonatal intestinal diseases. The second article by Tsigalou et al. reviews the association between peri-operative interventions and gut microbial community. While gastrointestinal surgeries have a significant impact on the epithelial barrier, the composition of the gut microbiome can influence surgical outcomes including complications like anastomotic leaks. The review highlights the current intervention studies with probiotics to address intestinal dysbiosis and reduce complications in surgical patients. A third review (Ye et al.) explores the connection between gut microbiome changes with kidney transplantation. Poor kidney function can disrupt the balance of intestinal microbiota, but administering prophylactic drugs to regulate the microbiota of patients may mitigate the occurrence and advancement of transplantation complications.

Two other review articles address chronic liver diseases in connection with gut microbiota with one exploring the use of plant natural products (Cai et al.) on metabolic-associated fatty liver disease (MAFLD) and the second one focusing on the relationship between *Helicobacter pylori* infection and nonalcoholic fatty liver disease (NAFLD) (Chen et al.). Both MAFLD and NAFLD, characterized by liver fat accumulation, lack approved drugs due to their complex nature. Recognizing the link between gut microbiota and MAFLD, there is growing interest in using plant natural products for its treatment. In Cai et al., the authors conduct a systematic review of the plant products that target the gut microbiota and show potential as safer and more effective treatments in the development of natural anti-MAFLD drugs. The second article focuses on the role of *H.pylori*, a gram-negative bacillus that has been suggested as an initiating factor for various diseases ranging from chronic gastritis, and Alzheimer’s to NAFLD. Chen et al., conducted a meta-analysis on the extra gastric role of *H.pylori* in connection with the development of NAFLD including alterations in inflammatory cytokines, insulin resistance, and lipid metabolism. Furthermore, another systematic review (Korczynska et al.) focuses on pathogenetic pathways that are shared by the intestinal microbiome and the uterus, highlighting the importance of studying microbiome modulations for the treatment of uterine fibroids.

Furthermore, there are two reviews on the oral-gut-circulatory axis highlight the bidirectional influence of immune cells, inflammatory factors, circulating bacteria, and microbial metabolites on the homeostasis of oral and gut microbiota (Tortora et al. and Kudra et al.). Both of these comprehensive reviews focus on recent studies on associations between oral microbiota to cancer susceptibility, particularly colon cancer. While targeting systemic inflammation may have therapeutic potential, the development of non-invasive screening tools like personalized medicine, probiotics, and lifestyle modifications may offer preventative approaches. Finally, delving into the research progress on intratumoral microbiota associated with bladder cancer, Lou et al., summarize the role of different bacterial phyla in cancer onset, progression, and prognosis. They highlight the challenges that continue to persist, including ethical and methodological issues, necessitating future investigation for clinical insights.

The compilation of the above review articles in this Research Topic comprehensively delves into a broad spectrum of gut microbiome research. It unveils fresh perspectives, assesses novel tools, and facilitates discussion on multiple dimensions of gut microbiome homeostasis. Furthermore, it explores diverse facets of its intricate association with various ailments and diseases, providing an examination of this dynamic field.

## Author contributions

SC: Writing – review & editing, Writing – original draft. LM: Writing – review & editing, Writing – original draft.

## References

[B1] Liu B.-N. Liu X.-T. Liang Z.-H. Wang J.-H. (2021). Gut microbiota in obesity. World J. Gastroenterol. 27, 3837–3850. doi: 10.3748/wjg.v27.i25.3837 34321848 PMC8291023

[B2] Tsai H.-J. Tsai W.-C. Hung W.-C. Hung W.-W. Chang C.-C. Dai C.-Y.. (2021). Gut microbiota and subclinical cardiovascular disease in patients with type 2 diabetes mellitus. Nutrients 13, 2679. doi: 10.3390/nu13082679 34444839 PMC8397936

[B3] Xiong R.-G. Li J. Cheng J. Zhou D.-D. Wu S.-X. Huang S.-Y.. (2023). The role of gut microbiota in anxiety, depression, and other mental disorders as well as the protective effects of dietary components. Nutrients 15, 3258. doi: 10.3390/nu15143258 37513676 PMC10384867

[B4] Xu H. Liu M. Cao J. Li X. Fan D. Xia Y.. (2019). The dynamic interplay between the gut microbiota and autoimmune diseases. J. Immunol. Res. 2019, 7546047. doi: 10.1155/2019/7546047 31772949 PMC6854958

